# Zoonotic and Anthropophilic *Trichophyton mentagrophytes* Complex Infection in Human: An Update and Narrative Review

**DOI:** 10.1111/myc.70082

**Published:** 2025-06-21

**Authors:** Settanan Plangsiri, Roberto Arenas, Teerapong Rattananukrom

**Affiliations:** ^1^ Faculty of Medicine Ramathibodi Hospital Mahidol University Bangkok Thailand; ^2^ Mycology Section “Dr. Manuel Gea Gonzalez” General Hospital Mexico City Mexico; ^3^ Division of Dermatology, Department of Medicine, Faculty of Medicine Ramathibodi Hospital Mahidol University Bangkok Thailand

**Keywords:** antifungal resistance, polymerase chain reaction, *T. mentagrophytes*, *Trichophyton indotineae*, *Trichophyton interdigitale*

## Abstract

**Background:**

*Trichophyton mentagrophytes* species complex comprises dermatophytes responsible for common superficial fungal infections affecting keratinised tissues. Recent shifts in taxonomy and increasing antifungal resistance—necessitate an updated understanding of this fungal complex.

**Objective:**

This narrative review provides a comprehensive update on the taxonomy, host immune response and clinical genotyping of the *T. mentagrophytes* complex, with a focus on zoonotic and anthropophilic infections in humans.

**Methods:**

A comprehensive literature search was conducted across PubMed and Scopus using MeSH terms and relevant keywords related to *T. mentagrophytes*, *T. interdigitale*, and *T. indotineae*. Eligible English‐language publications up to March 2025—including original research, case reports, reviews and guidelines—were included.

**Results:**

The *T. mentagrophytes* complex includes several genotypes with distinct transmission profiles. Genotypes III/III* are primarily zoonotic; genotype VII is sexually transmitted, especially in MSM populations; genotype VIII (*T. indotineae*) is anthropophilic and associated with terbinafine resistance. Infection involves keratinocyte adhesion, enzymatic skin barrier degradation and activation of pro‐inflammatory cytokines and antimicrobial peptides. Both innate and adaptive immunity, particularly Th1 and Th17 responses, are critical for fungal clearance, whereas chronic infections are associated with impaired T‐cell function and skewed Th2 responses.

**Conclusion:**

Emerging genotypes and drug resistance within the *T. mentagrophytes* complex pose increasing clinical challenges. Awareness of transmission patterns, immune evasion mechanisms and resistance profiles is essential for accurate diagnosis and effective management of dermatophytosis.

## 1 Introduction

Fungi are a diverse and abundant group of organisms that play essential ecological roles, particularly in decomposition and nutrient cycling [1, 2]. However, some species are pathogenic to humans, posing significant diagnostic and therapeutic challenges. Of the estimated 3 million fungal species globally, only about 150–300 are known to be pathogenic to humans [3, 4, 5]. Among these, *Trichophyton mentagrophytes* is a notable dermatophyte capable of zoonotic transmission through contact with animals [6, 7]. Infections commonly originate from pets such as rabbits and rodents, but dogs, cats, pigs and other animals have also been implicated [8, 9].

*T. mentagrophytes* was first identified in 1842 as a causative agent of beard infections, from which its name—derived from the Latin *mentagra*—originates. Initially classified as *Microsporon mentagrophytes*, it was later reclassified as *T. mentagrophytes*, the name currently in use [10]. This species is a well‐established cause of dermatophytosis, or ringworm—superficial fungal infections that affect keratinised tissues such as skin, hair and nails, but spare mucosal surfaces [11, 12]. Dermatophyte infections are among the most common skin conditions worldwide, with a lifetime risk estimated of 10%–20% [13, 14, 15, 16, 17, 18]. Clinically, these infections are referred to as ‘tinea’ and are classified based on the site of involvement.

For instance, tinea capitis, which affects the scalp, eyebrows and eyelashes, commonly presents with well‐defined scaling, alopecia, black‐dot alopecia or kerion formation. Tinea corporis and tinea faciei, involving the trunk and face, typically appear as annular, scaly patches with central clearing, a slightly raised erythematous border and well‐demarcated margins. Tinea barbae, affecting the beard area, presents with erythema, scaling and follicular pustules. Tinea manuum and tinea pedis, involving the hands and feet respectively, manifest as hyperkeratosis with erythema. Tinea cruris affects the groin and is often associated with pruritus, a burning sensation and pustules at the lesion margin. Tinea unguium involves the nails [11, 13, 19].

Prompt diagnosis and treatment of *T. mentagrophytes* infections are essential to prevent chronic lesions and complications such as deeper follicular involvement (e.g., Majocchi granuloma), particularly in immunocompromised individuals [20, 21, 22]. As diagnostic and therapeutic methods evolve, it is imperative for clinicians to stay updated on emerging patterns of infection and resistance to ensure effective disease management.

This review provides an updated overview of the *T. mentagrophytes* complex, focusing on its taxonomy, pathogenicity and host immune response, and genotyping—particularly zoonotic and anthropophilic infections in humans—to enhance clinical recognition and management of this pathogen.

## 2 Materials and Methods

A comprehensive literature search was performed using electronic databases including PubMed and Scopus. The search strategy combined Medical Subject Headings (MeSH) and free‐text keywords. The primary search terms included: ‘*Trichophyton mentagrophytes*’, ‘*Trichophyton interdigitale*’, ‘*Trichophyton indotineae*’, ‘dermatophyte’, ‘zoonotic dermatophytosis’, ‘anthropophilic dermatophyte’, ‘genotype’, ‘taxonomy’, ‘morphology’, ‘virulence factors’, ‘immune response’, ‘keratinocyte’, ‘molecular’, ‘antifungal resistance’ and ‘terbinafine resistance’.

Eligible studies included English‐language publications up to March 2025 comprising original research articles, case reports, reviews and relevant clinical or mycological guidelines. Exclusion criteria were non‐peer‐reviewed content, articles with insufficient methodological rigour or those focused exclusively on dermatophyte species outside the *T. mentagrophytes* complex.

Titles and abstracts were screened for relevance, and selected full‐text articles were critically reviewed. Additional references were identified through manual screening of bibliographies in key articles. Extracted data were synthesised narratively to highlight emerging genotypes, clinical presentations, host–pathogen interactions and resistance patterns of the *T. mentagrophytes* complex.

## 3 Morphology, Nomenclature and Genotypes

The *T. mentagrophytes* complex comprises pleomorphic fungi that exhibit both anamorphic (asexual) and teleomorphic (sexual) forms [23]. The term specifically refers to the anamorphic state and includes species such as *T. mentagrophytes*, *T. interdigitale, T. indotineae* and others. Several of these species have corresponding teleomorphic forms, including *Arthroderma simii*, *Arthroderma vanbreuseghemii* and *Arthroderma benhamiae* [8]. Historically, morphological classification led to separate names for the same species based on their life stages [24].

**FIGURE 1:**
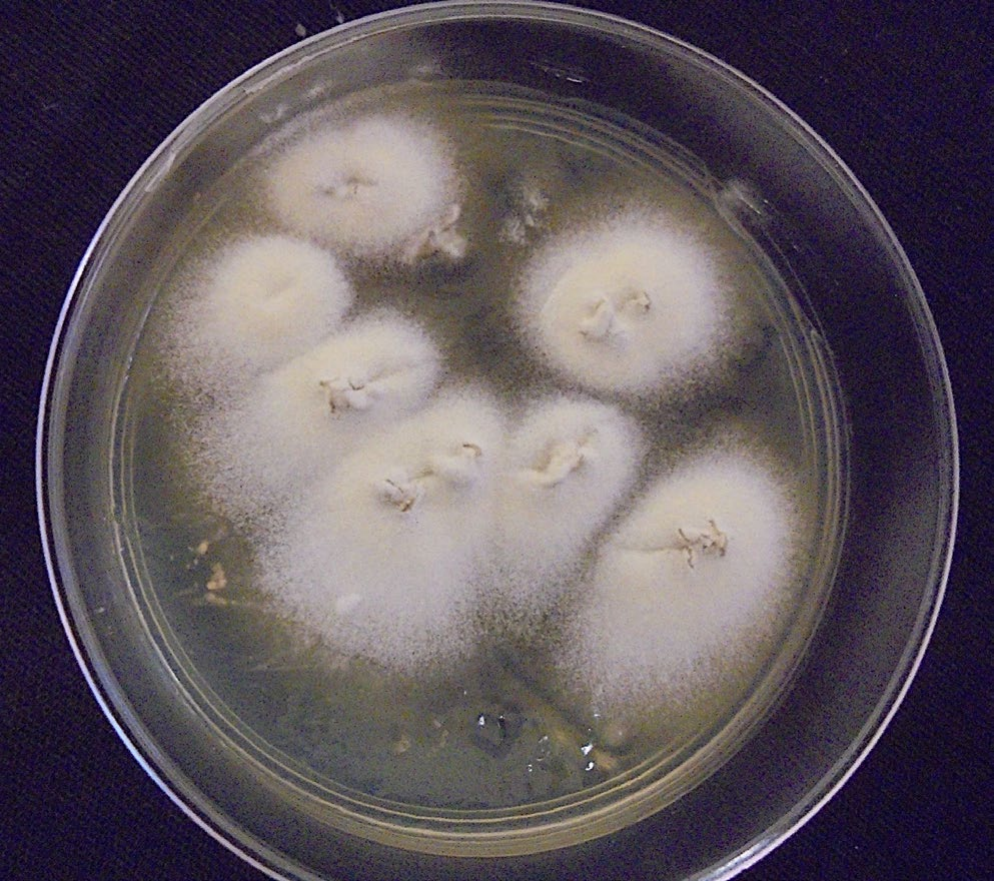
Colony morphology of *Trichophyton mentagrophytes* on Sabouraud dextrose agar, showing rapidly growing, powdery to granular, white colonies with a slightly raised, concentric surface and a cream to yellowish reverse. The colony appearance is characteristic of the *T. mentagrophytes* complex.

**FIGURE 2:**
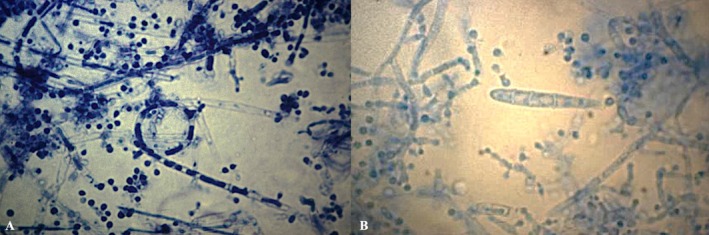
Lactophenol cotton blue preparation at 40× magnification showing microscopic features of *Trichophyton mentagrophytes* complex. (A) Septate hyphae with numerous spherical microconidia arranged in grape‐like clusters and spiral hyphae. (B) Similar findings with the presence of a characteristic cigar‐shaped, multicellular macroconidium.

When cultured on Sabouraud dextrose agar, colonies of the *T. mentagrophytes* complex appear flat, powdery and white, yellow or brown (Figure 1) [25]. Microscopically, they show branched, septate hyphae, sometimes with spiral forms. Club‐shaped macroconidia are rare, whereas unicellular microconidia are abundant (Figure 2) [6, 25].

Advances in molecular diagnostics have revealed that many previously distinct species were in fact different forms of the same organism. This led to the adoption of the ‘one fungus, one name’ initiative, which established *T. mentagrophytes* as the unified name for this group [26].

Molecular techniques have also enabled finer differentiation within the complex, leading to the recognition of distinct species such as *T. erinacei, T. quinckeanum, T. interdigitale, T. indotineae* and *T. mentagrophytes*. Within *T. interdigitale* and *T. mentagrophytes*, numerous genotypes have been identified—ranging from Type I to XII in *T. interdigitale* and Type I to XXVIII in *T. mentagrophytes* [24, 27, 28].

**FIGURE 3:**
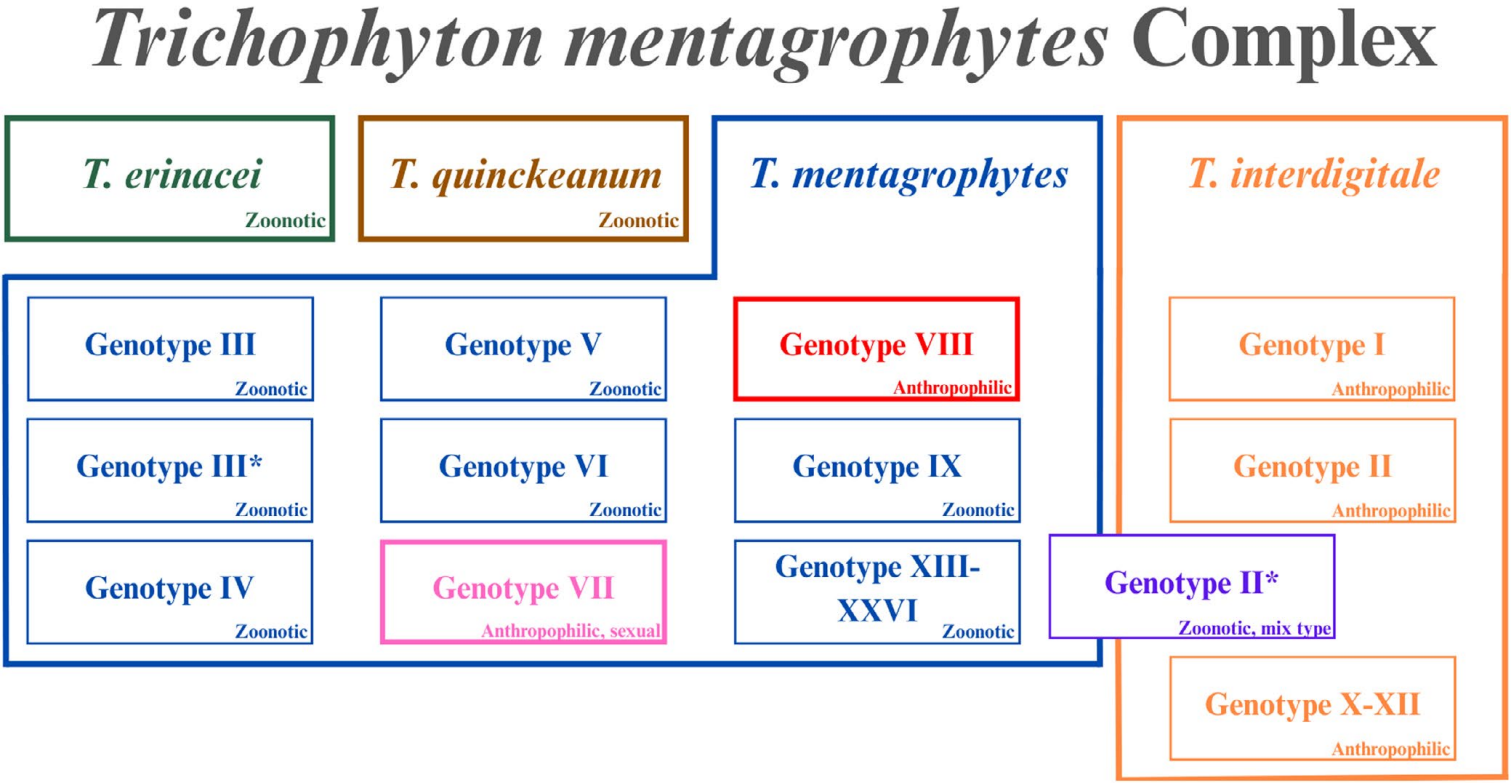
Genotypic diversity in the *Trichophyton mentagrophytes* complex. Each species within the complex is colour‐coded. Red and pink highlight anthropophilic genotypes within the predominantly zoonotic *T. mentagrophytes*. Purple indicates a mixed *T. mentagrophytes*/*T. interdigitale* genotype.

A 2018 reclassification distinguished *T. interdigitale* as anthropophilic (human‐associated) and *T. mentagrophytes* as zoonotic (animal‐associated). However, Genotype VIII of *T. mentagrophytes*, which exhibited anthropophilic characteristics and terbinafine resistance, was reclassified as a new species: *T. indotineae*. Genotype II, exhibiting overlapping features of both species, is considered a mixed type [27]. This reclassification has important clinical implications, particularly in understanding transmission dynamics and antifungal resistance patterns—especially the rising resistance observed in *T. indotineae* (Figure 3).

## 4 Host Immune Response

### 4.1 Skin Inoculation and Virulence Factors

Infection by the *T. mentagrophytes* complex begins when arthroconidia and microconidia come into contact with the skin. Arthroconidia express fibrils, whereas microconidia display carbohydrate‐specific adhesins, promoting strong adhesion to keratinised surfaces [29, 30, 31]. Adhesion strengthens over time, peaking between 3 and 6 h post‐contact, with some spores remaining attached for up to 12 h [30, 32, 33].

Between 4 and 24 h, the spores germinate and produce germ tubes that remain extracellular to corneocytes [33, 34]. By Day 3, fungal hyphae and mycelia invade the stratum corneum both perpendicularly and longitudinally, without penetrating keratinocyte [33, 34]. Electron microscopy reveals a distinctive ‘meandering’ hyphal growth pattern with increased mycelial branching as the infection deepens [31].

**FIGURE 4:**
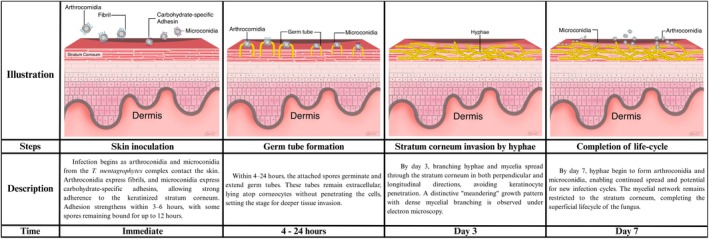
Stages of *Trichophyton mentagrophytes* complex infection in human skin. The diagram illustrates the fungal lifecycle from initial skin inoculation, germ tube formation and stratum corneum invasion to completion of the life cycle with spore production.

To facilitate invasion, *T. mentagrophytes* secretes various enzymes and toxins: SN‐38, a cytotoxic metabolite, induces keratinocyte apoptosis [35]; whereas metalloproteases (MEP), particularly MEP3 and MEP4, are highly expressed in this species [36]. Metalloproteases (MEP3 and MEP4) degrade extracellular matrix proteins [8]; elastase breaks down elastin to enhance skin penetration [37]. Other enzymes, including keratinase, collagenase, DNase, lipase and metallocarboxypeptidase, contribute to the degradation of keratin and structural skin components [31, 36, 38, 39]. The fungus further digests keratinocytes into peptides and amino acids, which serve as nutrients [40, 41]. By Day 7, hyphae begin to form arthroconidia, completing the infection cycle (Figure 4) [34].

Following invasion, the host immune system is rapidly activated. Keratinocytes initiate the response by recognising fungal components and signalling for immune engagement. The innate immune system—involving Langerhans cells, macrophages, neutrophils, mast cells and complement—acts to contain and eliminate the pathogen. In parallel, the adaptive immune response, particularly T cells, plays a central role in targeting and clearing fungal elements [42]. This coordinated immune activation is essential for controlling infection and restoring skin barrier integrity.

### 4.2 Keratinocyte Response

Keratinocytes infected with *T. mentagrophytes* exhibit marked cellular damage, including cytoplasmic oedema, expanded perinuclear spaces and plasma membrane disruption. Nucleoli become enlarged and displaced peripherally, whereas the rough endoplasmic reticulum shows membrane degradation. Mitochondria appear swollen with reduced cristae and weakened stroma. Additionally, lipid droplet aggregation and increased numbers of autophagic lysosomes are observed, in contrast to uninfected keratinocytes [43]. Notably, long non‐coding RNA expression in infected keratinocytes is significantly altered [44].

*T. mentagrophytes* infection induces a broad cytokine response in keratinocytes. This includes pro‐inflammatory cytokines (interleukin‐1β, IL‐6, IL‐6sR and IL‐17), immunomodulatory cytokines (IL‐7, IL‐15, IL‐16 and IP‐10), chemokines (IL‐8, MCP‐1, eotaxin and eotaxin‐2) and colony‐stimulating factors such as granulocyte colony‐stimulating factor and granulocyte‐macrophage colony‐stimulating factor. These responses are regulated via activation of the NF‐κB signalling pathway [35, 45, 46].

This cytokine milieu facilitates immune cell recruitment and amplifies the host response to infection. Histopathological examination of *T. mentagrophytes*‐infected epidermis typically reveals neutrophil predominance, indicative of an acute inflammatory response. In contrast, infections caused by *T. tonsurans* result in lower cytokine expression and a more chronic pattern, characterised by mononuclear cell infiltration and reduced neutrophilic activity [45, 46, 47, 48].

In addition to cytokines, keratinocytes secrete antimicrobial peptides (AMPs) as part of their innate defence. A key AMP is ACP5, a cationic peptide with potent antifungal activity against *T. mentagrophytes*. ACP5 demonstrates greater efficacy than fluconazole, lower cytotoxicity to human cells and a reduced likelihood of resistance development compared to terbinafine [49]. It also inhibits spore germination by inducing reactive oxygen species, leading to fungal membrane and mitochondrial damage and ultimately causing cellular deformation and death [49].

Other AMPs with antifungal activity against *T. mentagrophytes* include human β‐defensin‐2, ribonuclease 7 and psoriasin, with psoriasin showing the highest antifungal potency, possibly exceeding that of fluconazole [50]. Keratinocytes also upregulate cathelicidin (LL‐37) upon exposure to *T. mentagrophytes*, further inhibiting fungal proliferation [51].

IB‐367 is a synthetic AMP derived from protegrin‐1, a naturally occurring peptide found in porcine leukocytes exhibiting strong antifungal effects against *T. mentagrophytes* and has demonstrated synergistic activity when combined with standard antifungal agents such as fluconazole, itraconazole and terbinafine [52, 53]. Moreover, several novel AMPs—including allomyrinasin, andricin B, pinipesin, nigrocin‐HLM and Hs02—have also been shown to inhibit *T. mentagrophytes* growth in vitro [54].

### 4.3 Innate Immune Response

Langerhans cells, macrophages, neutrophils and mast cells are the primary innate immune cells recruited by keratinocytes in response to *T. mentagrophytes* infection [43]. Among these, Langerhans cells, a specialised subset of epidermal dendritic cells, play a particularly pivotal role. A reduction in their number has been correlated with increased susceptibility to superficial fungal infections [45, 55]. These cells express pathogen‐associated molecular patterns and pattern recognition receptors, including toll‐like receptors and C‐type lectin receptors, enabling pathogen recognition and immune activation [45, 56].

Specifically, the C‐type lectin receptor Langerin (CD207) binds fungal β‐glucans and mannans, facilitating fungal detection and immune signalling [57, 58]. Interestingly, in chronic *T. mentagrophytes* infections, Langerhans cells tend to accumulate in the epidermis, potentially reflecting prolonged immune stimulation [59]. As antigen‐presenting cells, they play a crucial role in bridging innate and adaptive immunity by promoting the activation of helper T cells and cytotoxic T cells [60, 61].

Macrophages and neutrophils are essential for fungal clearance. Upon dermatophyte invasion, these cells are recruited to the infection site through chemotactic signals [62]. Both cell types can phagocytose *T. mentagrophytes* arthroconidia even in the absence of opsonisation [63]. Neutrophils further contribute by releasing tumour necrosis factor‐alpha, neutrophilic granules and forming neutrophil extracellular traps, which immobilise and destroy fungal elements [64, 65].

The complement system enhances these defences by promoting opsonisation, facilitating chemotaxis, and forming the membrane attack complex, which directly lyses pathogens [66]. In *T. mentagrophytes* infections, both spores and hyphae have been shown to activate the complement cascade, underscoring its role in antifungal immunity [67].

### 4.4 Adaptive Immune Response

The adaptive cell‐mediated immune response against *T. mentagrophytes* is primarily mediated by T cells [68, 69]. Both CD4^+^ T‐helper cells and CD8^+^ cytotoxic T lymphocytes (CTLs) play essential roles in controlling the infection [70]. Among CD4^+^ subsets, T‐helper 1 (Th1) and T‐helper 17 (Th17) cells are particularly effective in mounting antifungal responses [71, 72]. Traditionally, the Th1 response—characterised by interferon‐gamma (IFN‐γ) production—was considered the dominant pathway. However, recent studies indicate that Th1 and Th17 (IL‐17‐producing) responses act synergistically to enhance fungal clearance [73]. This is further supported by clinical data showing that patients with chronic or recurrent *T. mentagrophytes* infections often exhibit reduced levels of Th1 (IFN‐γ^+^) and Th17 (IL‐17^+^) cells [72]. Additionally, elevated levels of regulatory T cells have been observed in infected patients, suggesting that Tregs may contribute to immune suppression and pathogen persistence [71].

In chronic infections, both T‐cell proliferation and cytotoxic activity are markedly impaired. In vitro studies using *T. mentagrophytes* mycelia and hyphae have demonstrated that CD4^+^ and CD8^+^ T‐cell function is significantly diminished in chronically infected individuals compared to healthy controls [70, 72]. Furthermore, chronic dermatophytosis is frequently associated with elevated serum IgE and IL‐4 levels, reflecting a skewed T‐helper 2 (Th2) response. This Th2 dominance is believed to impair effective fungal clearance and contribute to a persistent infection phenotype [71, 72, 74]. Individuals with atopic dermatitis or immediate‐type hypersensitivity appear particularly predisposed to chronic dermatophyte infections, including those caused by *T. mentagrophytes* [75, 76, 77].

Although IgG antibodies do not play a major role in fungal eradication, elevated IgG levels have been reported in both human and animal models of *T. mentagrophytes* infection [69, 72, 78, 79, 80, 81].

## 5 Reported Cases, Transmission Route and Animal Reservoir

It is hypothesised that dermatophytes originally evolved as geophilic organisms before adapting to animal hosts (zoonotic transmission) and ultimately developing into human‐adapted (anthropophilic) pathogens [82, 83]. The *T. mentagrophytes* complex has been implicated in a wide spectrum of tinea infections beyond tinea pedis; however, unlike *T. interdigitale*, it is typically not associated with onychomycosis [84]. Molecular characterisation of *T. mentagrophytes* infections has identified both anthropophilic genotypes—namely genotype VII and genotype VIII (*T. indotineae*)—as well as zoonotic and geophilic strains [28, 84, 85, 86, 87, 88, 89, 90, 91, 92, 93, 94]. All reported cases discussed below were confirmed by internal transcribed spacer (ITS) sequencing (Tables 1 and 2).

**TABLE 1:** *Trichophyton mentagrophytes* complex genotypes—transmission, reservoirs and clinical presentations.

Genotype	Transmission route	Host/Reservoir	Clinical presentations	Notes
III (snow leopard)/III* (Germany type 1)	Zoonotic	Snow leopards, cats, rabbits, dogs, chinchilla	Tinea barbae, kerion celsi, tinea corporis	Case in zookeeper's daughter; animal‐associated; genotype III* linked to higher human infection rate
V	Zoonotic	Sheep; occasional other animals	Tinea corporis, tinea faciei	Strong animal association; sporadic human cases
VII	Sexual (anthropophilic)	Humans (MSM); 1 case in cat	Deep pubogenital tinea, anogenital tinea	MSM‐associated outbreaks; 16‐case cluster from single masseur; called ‘Thai variant’; no terbinafine resistance
VIII (*T. indotineae*)	Human‐to‐human and sexual	Humans; reported in dogs	Widespread tinea corporis, cruris; resistant cases may be extensive and recalcitrant	Major concern due to terbinafine resistance (11.7% in India, 18.6% in North America); anthropophilic with suspected zoonotic reservoir
IX	Possibly environmental	Humans, soil	Tinea corporis, unspecified	Known as the ‘Australian strain’; limited clinical data; possible environmental reservoir

**TABLE 2:** *Trichophyton mentagrophytes* (TM) and *Trichophyton interdigitale* (TI) genotypes, geographic distribution and host species along with their corresponding GenBank codes [28, 84, 87, 88, 89, 90, 91, 92, 93, 94, 95].

Genotype	GenBank ITS codes	Geographic distribution	Host species	Clinical presentations
TI‐I	FM986691, KC595991	Europe, Australia, Iran, Tunisia	Human (anthropophilic)	Tinea pedis, tinea corporis, tinea unguium
TI‐II	JX122216, KP308373	Global	Human (anthropophilic)	Tinea pedis, tinea corporis, tinea unguium
TM‐II*	KP132819, MT858876, MT858877	Europe, Asia, Australia	Not specified	Tinea capitis, tinea faciei
TM‐III	AF506034, AB458171, KJ606099, KT253559, FM986750, MT858878, MK450325, KX866689, KJ606099	Europe, Japan, USA	Human (prevalent in children), cats, dogs, rabbits, chinchilla, soil	Tinea corporis, tinea capitis, tinea faciei
TM‐III*	FM986758, LN736306, MF926358, MT858923, JQ407193, MW346113, MK447605, MK447604	Europe, Canada, Japan, India	Human (prevalent in children), animals	Tinea capitis, tinea faciei, tinea barbae, tinea corporis, tinea genitalis, kerion celsi
TM‐IV	FM986773, KJ606102, MT858935, KJ722759, GU929694, MW346154, MK447609	Europe, USA, Japan, South Africa	Human, chinchilla, mouse	Tinea corporis
TM‐V	KJ606098, KU496915, MT374269, MT374268, MT374257, MT374258	Iran, Egypt, Iraq, Japan, Spain	Sheep	Tinea capitis, tinea faciei, tinea corporis, tinea cruris
TM‐VI	KT285210	Europe (Russia, Finland), Japan	Not specified	Tinea capitis
TM‐VII	JN134101, KT253558, MT858938, MK447611, MK450324, MK450322, MK450323	Europe, Australia, SEA, Iran, Egypt	Human (sexual transmission)	Tinea genitalis, tinea faciei, tinea cruris, tinea glutealis, tinea corporis, tinea barbae
TM‐VIII (*T. indotineae*)	JN133999, KY761968, KT192500, MT858945, MH791419, MH791420, MH791422, MN831065, MN831089, MN831064, MN831088	Europe, India, Iran, Japan, Africa	Human, canine	Tinea glutealis, tinea cruris, tinea corporis, tinea pseudoimbricata, tinea faciei, tinea manuum
TM‐IX	MK447613, MW346065, MW346048, MW346050	Australia, Germany, China	Human	Not available
TI‐X – TI‐XII, TI‐XIII – TI‐XXVI	MK312735, MT858875, MK312755, MF109039, MK312917, MK312950, MK312937, MK312933, MK312990, MK313028, MK312878, MK313030, MK312891, MK312888, AF170453, MT858874, MT858956	Mostly Iran, Europe, Japan	Mostly Human (limited data)	Not available

### 5.1 *T. mentagrophytes* Genotype III and III*

*T. mentagrophytes* genotypes III (Snow leopard) and III* (Germany type 1) are among the most prevalent in Europe and are primarily associated with zoonotic transmission [84, 90]. Infections caused by genotype III* have been reported to induce moderate to severe inflammatory lesions, whereas those caused by genotype III are typically milder. Both strains, however, show a preference for the facial region, with the highest incidence documented in cases of tinea capitis et faciei, followed by tinea corporis [84]. A well‐documented case involved the daughter of a zookeeper who contracted dermatophytosis from snow leopards exhibiting facial alopecia; transmission likely occurred during tick removal [91]. Another case in Germany described a girl with kerion celsi and her father with tinea barbae, both infected with *T. mentagrophytes* genotype III*, as confirmed by sequencing. However, the exact source of infection remained unclear, despite potential exposure to domestic cats and rabbits [96]. A separate report described a 3‐year‐old boy with kerion celsi caused by genotype III* as well [84]. Additionally, in two other genotype III* cases, the patients' cats were also found to be infected, suggesting zoonotic transmission [84]. Lastly, an infection with genotype III was identified in a 14‐year‐old girl presenting with lesions on her thigh [84]. While human infections have been reported, genotypes III and III* are predominantly isolated from niche animal hosts, supporting a zoonotic origin. Notably, genotype III* has been linked to a higher prevalence of tinea infections in humans [84, 87].

### 5.2 *T. mentagrophytes* Genotype VII

Recent reports highlight a rising number of infections caused by *T. mentagrophytes* genotype VII, particularly through sexual transmission among men who have sex with men (MSM) in European countries [97, 98, 99, 100, 101]. Genotype VII is typically associated with widespread moderate to severe inflammatory lesions, most commonly affecting the pubogenital region. In some cases, extensive ulceration has also been reported [84, 86]. Additional clinical manifestations linked to genotype VII include tinea faciei, barbae, genitalis, inguinalis, glutealis and corporis [98, 99, 100, 101]. Most cases involve individuals with multiple sexual partners, some of whom have also tested positive for other sexually transmitted diseases (STDs), including human immunodeficiency virus and monkey pox [97]. Infections typically affect the anogenital region, often presenting as deep pubogenital tinea [85, 102]. A notable outbreak involved a single masseur who transmitted the infection to 16 individuals, raising concerns about potential clusters of genotype VII infections [97]. Additional cases have been reported among travellers returning from Egypt and Southeast Asia following sexual encounters, suggesting broader geographic distribution [85, 86]. Due to its apparent regional association, some researchers have informally referred to this strain as the ‘Thai variant’ [103]. However, some cases of *T. mentagrophytes* genotype VII have been reported in individuals with no history of travel to Asian countries or identifiable risk factors for STDs. This may suggest undetected community transmission, population migration or undisclosed patient history. Although sexual transmission is the primary route, one isolate of genotype VII has been recovered from a domestic cat [98]. Importantly, unlike *T. indotineae*, no terbinafine‐resistant strains of genotype VII have been reported to date [98].

### 5.3 *T. mentagrophytes* Genotype VIII (*T. indotineae*)

**FIGURE 5:**
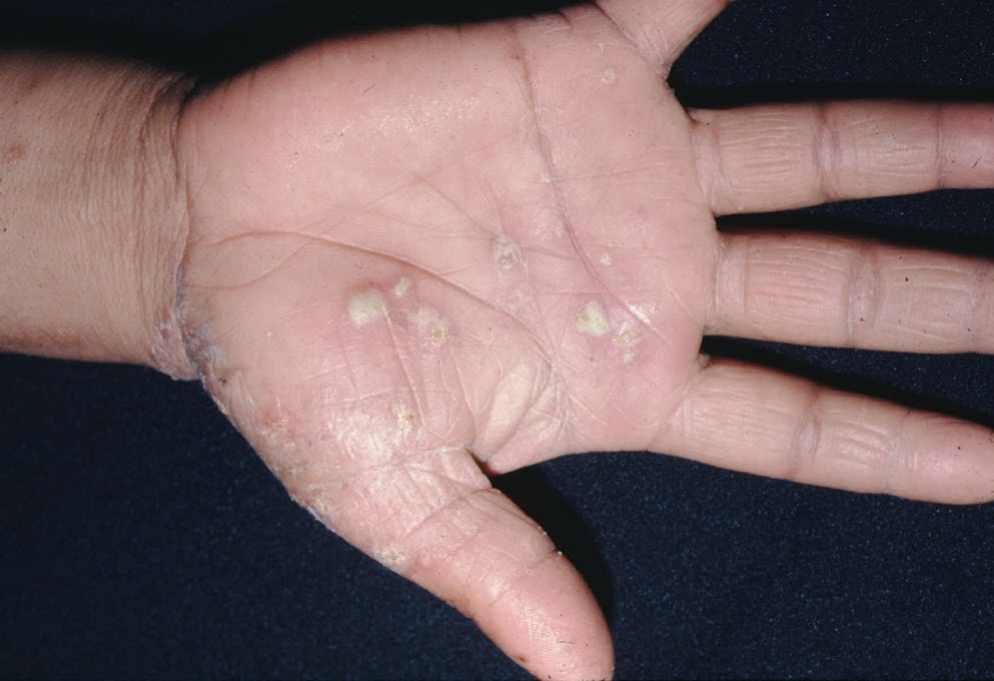
Inflammatory tinea manuum caused by *Trichophyton mentagrophytes complex*, presenting as well‐demarcated erythematous plaques with vesiculopustular lesions and scaling on the palmar surface.

*T. mentagrophytes* genotype VIII, also known as *T. indotineae*, has emerged as a significant public health concern due to increasing terbinafine resistance. Patients infected with *T. indotineae* are generally older compared to those infected with other strains. This genotype tends to target the gluteal and inguinal regions and is associated with the longest duration of infection [84]. Clinical suspicion of *T. indotineae* should arise in patients presenting with chronic, extensive and atypical dermatophytic lesions, particularly when characterised by erythematous, scaly concentric plaques with a classic ‘ring‐on‐ring’ or pseudoimbricata appearance, along with papulosquamous or pustular features. The involvement often includes the groin, gluteal cleft and scrotum, and is frequently associated with intense pruritus. A history of over‐the‐counter topical corticosteroid use, especially in combination creams, may result in steroid‐modified tinea with an altered clinical presentation. Importantly, a lack of clinical response to a 4‐week course of oral terbinafine, and often to standard topical antifungals as well, further supports suspicion of *T. indotineae* infection. Other commonly affected sites include tinea corporis [84, 90, 104], tinea faciei [104], tinea manuum (Figure 5) and tinea cruris [90, 104]. Notably, there has also been a report suggesting sexual transmission of genotype VIII [104]. Resistant strains have been reported across Asia, Europe and Africa [82], with resistance rates estimated at 11.7% in India and 18.6% in North America [105, 106]. The primary genetic factor associated with allylamine resistance in *T. indotineae* is mutation of the squalene epoxidase (SQLE/ERG1) gene. These mutations typically involve single or multiple amino acid substitutions, most commonly at Leu393, Phe397, Phe415 and His440. The most frequently reported variants are Phe397Leu and Leu393Phe. Notably, the Phe397Leu mutation, frequently observed in *T. indotineae*, has also been reported in *T. mentagrophytes* genotype VII, underscoring its role in antifungal resistance. Given the subtlety of these single‐nucleotide changes, sequencing is essential for detecting resistance‐associated mutations, similar to ITS sequencing for species identification. Beyond identifying resistant strains, SQLE sequencing is critical for epidemiological tracking, as shown by the detection of Phe397Leu and Leu393Phe mutations in both India and Germany, suggesting a shared origin. Likewise, the Leu393Ser mutation has been documented in France and Bangladesh. Both human‐to‐human and sexual transmission have been documented [104]. It is hypothesised that this genotype has adapted to human skin, leading to its classification as anthropophilic [82, 83]. However, *T. mentagrophytes* genotype VIII has also been isolated from animals such as dogs, suggesting the existence of zoonotic reservoirs that may contribute to its pathogenic persistence and transmission [87, 107]. In a systematic review and meta‐analysis of 27 studies comprising 81 cases, the report showed that *T. indotineae* infections affected both sexes equally. Prior topical steroid use (25%) was significantly associated with treatment failure, and terbinafine resistance was observed in 85.3% of cases. Oral itraconazole was significantly linked to clinical cure, with a median time to complete resolution of 11.5 weeks and a recurrence rate of 19.7% [108].

### 5.4 Other *T. mentagrophytes* Genotypes

Additional genotypes include genotype IX, also known as the ‘Australian strain’, and genotype V, which has been primarily isolated from sheep in Iran. Sporadic infections involving humans and other animals suggest that sheep may serve as a reservoir for genotype V [109]. Genotype V infections in humans are most commonly associated with tinea corporis and tinea cruris [90]. Genotype II* has been reported to cause both tinea capitis and tinea corporis, whereas genotype IV has been linked to cases of tinea corporis and tinea pedis [84]. Several other genotypic variants of *T. mentagrophytes* have been identified and deposited in public databases, though they are currently considered to have limited clinical or epidemiological relevance.

## 6 Conclusion

This review highlights key developments in the understanding of zoonotic and anthropophilic infections caused by the *T. mentagrophytes* complex. Taxonomic reclassification, especially the identification of *T. indotineae*, underscores the importance of molecular tools in species and genotype identification. Genotype III/III*: predominantly zoonotic, linked to animal reservoirs (e.g., snow leopards, cats, rabbits), and common in Europe; Genotype VII: recently emerged as a sexually transmitted pathogen, particularly among MSM in Europe; Genotype VIII (*T. indotineae*): of public health concern due to widespread terbinafine resistance and human‐to‐human transmission.

The pathogenesis involves initial spore adhesion to the stratum corneum, followed by enzymatic degradation and immune activation. Keratinocytes produce cytokines and AMPs (e.g., ACP5, psoriasin, LL‐37), triggering recruitment of innate immune cells. Adaptive immunity, particularly Th1 and Th17 responses, is vital for fungal clearance. Chronic infections correlate with immune suppression, heightened Th2 responses and elevated IgE.

Clinicians should remain vigilant regarding evolving genotypes, atypical clinical presentations and antifungal resistance to ensure timely diagnosis and appropriate treatment of *T. mentagrophytes*‐related infections.

## Author Contributions

**Settanan Plangsiri:** visualization, writing – original draft, writing – review and editing, resources, conceptualization, data curation, investigation. **Roberto Arenas:** supervision, resources, visualization. **Teerapong Rattananukrom:** writing – review and editing, visualization, supervision, conceptualization.

## Conflicts of Interest

The authors declare no conflicts of interest.
